# Metastable Reprogramming State of Single Transcription Factor-Derived Induced Hepatocyte-Like Cells

**DOI:** 10.1155/2019/6937257

**Published:** 2019-04-07

**Authors:** Seon In Hwang, Tae Hwan Kwak, Ji Hyun Kang, Jonghun Kim, Hyunseong Lee, Kee-Pyo Kim, Kinarm Ko, Hans R. Schöler, Dong Wook Han

**Affiliations:** ^1^Department of Stem Cell Biology, School of Medicine, Konkuk University, 120 Neungdong-ro, Gwangjin-gu, Seoul 05029, Republic of Korea; ^2^Department of Cell and Developmental Biology, Max Planck Institute for Molecular Biomedicine, Röntgenstraße 20, 48149 Münster, Germany; ^3^KU Open-Innovation Center, Institute of Biomedical Science & Technology, Konkuk University, 120 Neungdong-ro, Gwangjin-gu, Seoul 05029, Republic of Korea; ^4^Department of Advanced Translational Medicine, School of Medicine, Konkuk University, 120 Neungdong-ro, Gwangjin-gu, Seoul 05029, Republic of Korea

## Abstract

We previously described the generation of induced hepatocyte-like cells (iHeps) using the hepatic transcription factor *Hnf1a* together with small molecules. These iHeps represent a hepatic state that is more mature compared with iHeps generated with multiple hepatic factors. However, the underlying mechanism of hepatic conversion involving transgene dependence of the established iHeps is largely unknown. Here, we describe the generation of transgene-independent iHeps by inducing the ectopic expression of *Hnf1a* using both an episomal vector and a doxycycline-inducible lentivirus. In contrast to iHeps with sustained expression of *Hnf1a*, transgene-independent *Hnf1a* iHeps lose their typical morphology and *in vitro* functionality with rapid downregulation of hepatic markers upon withdrawal of small molecules. Taken together, our data indicates that the reprogramming state of single factor *Hnf1a-*derived iHeps is metastable and that the hepatic identity of these cells could be maintained only by the continuous supply of either small molecules or the master hepatic factor *Hnf1a*. Our findings emphasize the importance of a factor screening strategy for inducing specific cellular identities with a stable reprogramming state in order to eventually translate direct conversion technology to the clinic.

## 1. Introduction

Somatic cell fates determined during development can be reversed into an embryonic stem cell-like state by the forced expression of *Oct4*, *Klf4*, *Sox2*, and *cMyc* (i.e., OKSM), resulting in the generation of induced pluripotent stem cells (iPSCs) [1, 2]. Converting a differentiated state into cellular pluripotency is a highly orchestrated process in which both exogenous OKSM factors and their endogenous counterparts play a distinct role in a stage-specific manner [3–5]. For initiating the reprogramming process, each reprogramming factor plays an essential and distinct role, such as erasing somatic identity and activating the endogenous counterpart. During the reprogramming process, exogenous reprogramming factors, in cooperation with their activated endogenous counterparts, drive the pluripotential state of iPSCs by remodeling chromatin structures and subsequently recruiting pluripotency-associated factors to their target loci [6, 7]. After the successful reprogramming of differentiated cells into an iPSC state, the transgenes are typically silenced due to high levels of DNA methyltransferases in iPSCs [3]. This result indicates that the transgenes are dispensable in the maintenance of an iPSC state [8] and that the endogenous pluripotential network is actually sufficient for maintaining cellular pluripotency in iPSCs without the assistance of any transgenes [3, 8].

Recent studies have also demonstrated that cell type-specific transcription factors, together with specific culture conditions, could also confer distinct cellular identities onto somatic cells [9–31]. The directly converted cell types exhibit key cellular and functional features of their *in vivo* counterparts [9–31]. Previous studies [32, 33] have also attempted to elucidate the role of transdifferentiation factors in the process of direct conversion into neurons and cardiomyocytes. However, the role of hepatic reprogramming factors in the generation of induced hepatocyte-like cells (iHeps) remains largely unknown. We previously described that the hepatic conversion process is a step-wise transition in which distinct molecular and cellular events occur in a sequential manner and that *Hnf1a* alone could induce somatic cells to adopt a mature hepatic identity [31]. More recently, we have also demonstrated that *Hnf1a*, together with *Foxa3*, could drive the robust direct generation of induced hepatic stem-like cells (iHepSCs), which could be also converted into cholangiocyte progenitor cells by the Notch signal [10]. Our data indicates that exogenous *Hnf1a* is indeed a master hepatic factor that could confer either a mature hepatic state or even a more progenitor state onto somatic cells. However, the role of this factor following the successful conversion into the hepatic state remains elusive.

In this study, we attempted to decipher the role of exogenous *Hnf1a* in the hepatic conversion process by controlling its expression using transgene controllable reprogramming systems such as an episomal vector or a doxycycline-inducible lentivirus. In contrast to iHeps with sustained expression of exogenous *Hnf1a*, transgene-independent iHeps generated by either episomal vector or doxycycline-inducible lentivirus encoding *Hnf1a* could not be maintained stably in culture, as they rapidly lost their typical hepatic features upon withdrawal of small molecules. However, iHeps generated by multiple hepatic factors (*Hnf4a* and *Foxa3*; *Gata4*, *Hnf1a*, and *Foxa3*) could be maintained stably, even in the absence of both sustained transgene expression and small molecules. Collectively, our data indicates that the reprogrammed state of iHeps generated by *Hnf1a* alone is metastable and that the continuous expression of exogenous *Hnf1a* or small molecules is required for stabilizing this metastable state. Our findings provide evidence for a reprogramming protocol producing a metastable cellular state that should be stabilized for the translation of this direct conversion technology to the clinic. Thus, for further translation of direct conversion technology, we should screen reprogramming cocktails for inducing not only a robust cell fate conversion but also a stably reprogrammed cellular identity.

## 2. Materials and Methods

### 2.1. Cell Culture

Mouse primary hepatocytes were isolated from the liver tissue of 8-week-old C57/B6 mice by the traditional collagenase perfusion protocol [34]. Primary hepatocytes, e-iHeps, and r-iHeps were maintained in hepatocyte culture medium (HCM), consisting of DMEM/F-12 (Invitrogen) supplemented with 10% fetal bovine serum (FBS) (Seradigm), 0.1 *μ*M dexamethasone (Sigma), 10 mM nicotinamide (Sigma), 1% insulin-transferrin-selenium (ITS) premix (Gibco), 1% penicillin/streptomycin (PS) (Gibco), GlutaMAX™ (Gibco), 10 ng/ml fibroblast growth factor 4 (FGF4) (Peprotech), 10 ng/ml hepatocyte growth factor (HGF) (Peprotech), and 10 ng/ml epidermal growth factor (EGF) (Peprotech). Freshly isolated mouse primary hepatocytes were cultured onto gelatin-coated dish and collected after 48 hrs for RNA extraction. Mouse embryonic fibroblasts (MEFs) were isolated through single-cell dissociation of E13.5 C57/B6 mouse embryos after removing the head and all the internal organs, including the liver and intestine. Isolated MEFs were cultured in MEF medium (MEFM), composed of DMEM high glucose (Biowest), 10% FBS (Seradigm), 1% MEM/NEAA (Gibco), 1% penicillin/streptomycin (PS) (Gibco), and GlutaMAX™ (Gibco).

### 2.2. Establishment of Gene Delivery Systems

For establishing distinct gene delivery systems, we amplified the CDS region of the hepatic reprogramming factors Hnf1a, Hnf4a, Gata4, and Foxa3 by Phusion High-Fidelity DNA Polymerase (Thermo Scientific). Amplified PCR products were inserted into the gateway cloning donor vector pCR8/GW/TOPO (Invitrogen) according to the manufacturer's instructions. The cloned CDS regions in the donor plasmids were transferred into the gateway destination plasmids by LR recombination. The gateway destination plasmids pCXLE-gw (Addgene #37626), pMXs-gw (Addgene #18656), and FU-tetO-gw (Addgene #43914) were used for the episomal, retroviral, and dox-inducible lentiviral gene delivery systems, respectively. For preparing transfection-quality plasmid DNA, cloned plasmid DNA was amplified and purified by the Genopure Plasmid Maxi Kit (Roche).

### 2.3. Generation of iHeps

To generate e-iHeps, 1.0 × 10^6^ MEFs were transfected with 5.0 *μ*g of the episomal vector using the Amaxa P4 Primary Cell 4D-Nucleofector kit (Lonza) according to the manufacturer's instructions. A total of 1.5 × 10^5^ transfected cells were plated onto collagen-coated 35 mm cell culture dishes and cultured in MEFM for 48 hrs [13]. After 48 hrs of transfection, the cells were cultured in HCM with the small molecules A83-01, BMP4, and CHIR99201 (ABR). 1a e-iHeps can be expanded stably over 20 passages in the presence of ABR. For r-iHep generation, the MEFs were transduced with retroviral particles and cultured as previously described [31]. Briefly, 5 × 10^4^ MEFs were seeded onto 0.1% gelatin-coated 35 mm cell culture dishes and incubated with retrovirus-containing MEFM together with 8 *μ*g/ml of protamine sulfate (Sigma) for 48 hrs. After 48 hrs of viral infection, the medium was replaced with HCM containing ABR. To generate d-iHeps, 5 × 10^4^ cells of MEFs were seeded onto 0.1% gelatin-coated 35 mm cell culture dishes and incubated with dox-inducible lentivirus-containing MEFM together with 8 *μ*g/ml of protamine sulfate (Sigma) for 48 hrs. After 48 hrs, the medium was replaced with HCM containing 1 *μ*g/ml of doxycycline (Tocris). The culture medium was changed every other day.

### 2.4. Gene Expression Analysis

To compare the relative expression level of marker genes, the total RNA was isolated from the cell pellet using the Hybrid-RTM RNA isolation kit (GeneAll). Complementary DNA (cDNA) was synthesized with the High Capacity cDNA Reverse Transcription kit (Applied Biosystems) using 1 *μ*g of isolated total RNA. Quantitative RT-PCR (qPCR) was performed with the SYBR green PCR Master Mix (Applied Biosystems) using the ABI 7500 real-time PCR system (Applied Biosystems). ΔCt values were calculated by subtracting the Gapdh Ct value from that of each target gene. Relative expression levels were calculated by using the 2^−ΔΔCt^ method. Primer sequences used for qPCR are listed in supplementary Table S1.

### 2.5. Immunocytochemistry

Immunocytochemistry was performed as we described previously [35]. Briefly, cells were fixed with 4% paraformaldehyde (Sigma) for 20 min at room temperature and washed three times with PBS. Fixed cells were then permeabilized and blocked with DPBS (Welgene) containing 0.03% Triton X-100 (Sigma) and 5% FBS (Seradigm) for 1 hr at room temperature. Permeabilized cells were then incubated with a primary antibody mixture for 16 hrs at 4°C and incubated with the appropriate secondary antibody after washing three times. Counterstaining was performed with Hoechst 33342 (Sigma). Primary antibodies used for immunocytochemistry are as follows: mouse anti-albumin (R&D Systems, 1 : 100), rabbit anti-*α*-1-antitrypsin (Abcam, 1 : 100), mouse anti-CK18 (Abcam, 1 : 200), rabbit anti-E-cadherin (Cell Signaling, 1 : 200), and rabbit anti-ZO1 (Invitrogen, 1 : 200).

### 2.6. Dil-Ac-LDL Assays

To measure the LDL uptake ability of the reprogrammed e-iHeps, cells were incubated with 10 *μ*g/ml acetylated LDL labeled with 1,1′-dioctadecyl-3,3,3′,3′-tetramethylindo-carbocyanine perchlorate (Dil-Ac-LDL) (Invitrogen) for 4 hrs in a CO_2_ incubator. Nuclei were stained with Hoechst 33342. The Zeiss Axiovert 200 inverted fluorescence microscope equipped with an AxioCam HRm camera was used for acquiring images.

### 2.7. PAS Staining and the ICG Uptake Assay

The Periodic acid-Schiff kit (Invitrogen) was used for Periodic acid-Schiff (PAS) staining according to the manufacturer's instructions. Briefly, cells were fixed with 10% formalin in 95% cold ethanol and rinsed slowly with running tap water for 1 min. Fixed cells were exposed to periodic acid solution for 5 min at room temperature and washed three times with distilled water. The cells were exposed to Schiff's reagent for 15 min at room temperature and washed three times with tap water for 5 min. For the indocyanine green (ICG) uptake assay, cells were incubated with HCM containing ICG solution (Sigma) in a CO_2_ incubator (37°C) for 1 hr and washed three times using PBS. Images were acquired using an Olympus CKX41 microscope with a Canon EOS 600D camera.

### 2.8. Albumin Secretion Assay

To measure the amount of secreted albumin in the culture medium, supernatants from the different cell types (MEFs, iHeps, and primary hepatocytes) were collected after 48 hrs of culture and analyzed using the Mouse Albumin ELISA Kit (Bethyl Laboratories) according to the manufacturer's instructions.

### 2.9. Urea Secretion Assay

The amount of urea in the culture media was measured in a time-course manner after the addition of 1 mM ammonium chloride (Sigma) to cultures of MEFs, iHeps, and primary hepatocytes. The urea was detected using the QuantiChrom Urea Assay Kit (BioAssay Systems) according to the manufacturer's instructions.

### 2.10. Copy Number Detection

To analyze the copy number of the episomal vector in e-iHeps, a serially diluted plasmid vector and genomic DNA were used for generating a standard curve. The copy number was determined by the Ct value of EBNA-1, and the cell number was determined by the Ct value of Fbxo15. Primer sequences are described in supplementary Table S1.

### 2.11. Flow Cytometry

For flow cytometry analysis, cells were dissociated into a single-cell suspension with trypsin and washed twice with PBS. Cells were fixed at 4% paraformaldehyde (Sigma) for 20 min. After washing two times with PBS, fixed cells were incubated with blocking solution containing 0.03% Triton X-100 (Sigma) and 5% FBS (Seradigm) for 10 min. The cells were incubated with the primary antibody for 30 min at 4°C. After washing two times with PBS, cells were incubated with the appropriate fluorescence-conjugated secondary antibody for 20 min in the dark at 4°C. Analysis was done by using a BD Accuri™ (BD Biosciences). Primary antibodies used for flow cytometry are as follows: rabbit anti-E-cadherin (Cell Signaling, 1 : 200) and goat anti-albumin (Bethyl Laboratories, 1 : 200).

### 2.12. Statistical Analysis

For statistical analysis, the unpaired *t*-test was used for calculating *P* values. All the values are from at least triplicated analysis, and the *P* values are presented as ^∗^ *P* < 0.05, ^∗∗^ *P* < 0.01, and ^∗∗∗^ *P* < 0.001.

## 3. Results

### 3.1. Generation of Integration-Free iHeps Using Hnf1a

**Figure 1:**
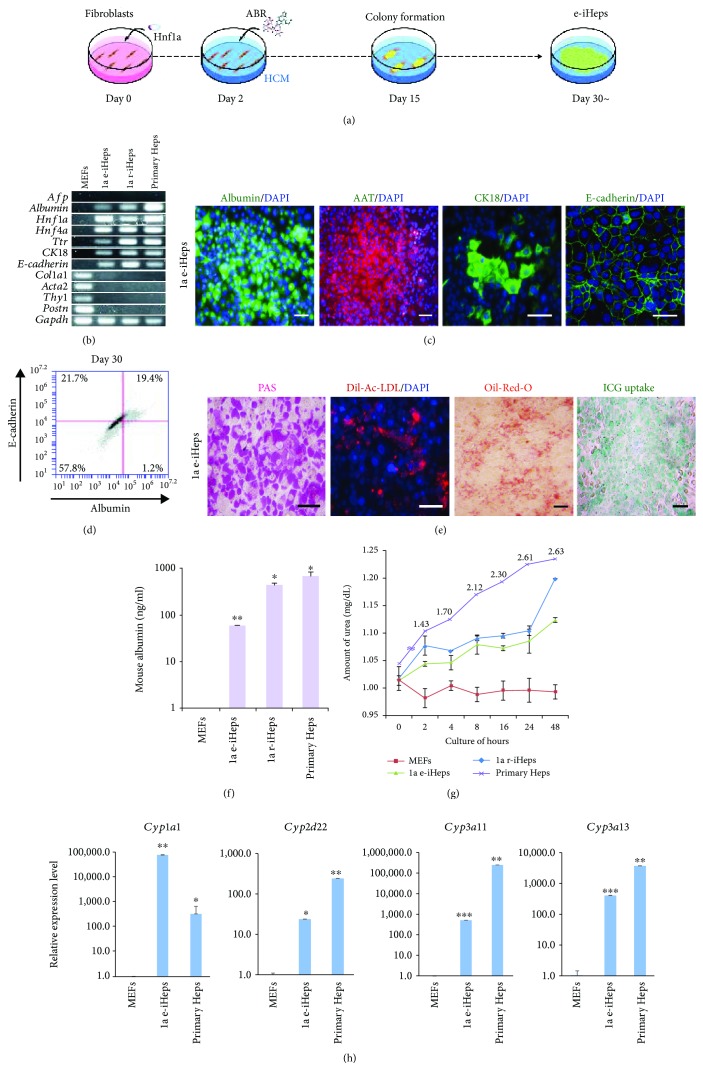
Generation of integration-free iHeps using *Hnf1a*. (a) Schematic diagram depicting the procedure of e-iHep generation using an episomal vector encoding *Hnf1a*. (b) Gene expression patterns of hepatocyte- and fibroblast-specific markers in e-iHeps and r-iHeps were analyzed by RT-PCR. (c) Fluorescence microscopy images of e-iHeps immunostained with antibodies raised against albumin, Aat, CK18, and E-cadherin. Scale bars: 100 *μ*m. (d) Flow cytometry analysis describing the percentage of albumin (*x*-axis) and E-cadherin (*y*-axis) double-positive cells on day 30 after transfection. (e) Functional analysis of e-iHeps including Periodic acid-Schiff (PAS) staining, intake of acetylated low-density lipoprotein (Ac-LDL), Oil-red-O staining, and indocyanine green (ICG) uptake. Scale bars: 100 *μ*m. (f and g) Both albumin secretion (f) and urea production (g) were determined in e-iHeps. MEFs and primary hepatocytes were used as negative and positive controls, respectively. Error bars indicate the standard deviation of triplicate values. ^∗^ *P* < 0.05 and ^∗∗^ *P* < 0.01. (h) Expression levels of CYP450 genes in e-iHeps were analyzed by qPCR upon treatment of CYP inducers (3-methylcholanthrene, rifampicin, and dexamethasone). Expression levels were normalized to those of MEFs. Error bars indicate the standard deviation of triplicate values. ^∗^ *P* < 0.05, ^∗∗^ *P* < 0.01, and ^∗∗∗^ *P* < 0.001.

We previously described that the hepatic factor *Hnf1a* could convert mouse somatic cells into induced hepatocyte-like cells (iHeps), which represent cells of a more mature hepatic state compared with iHeps generated by hepatic reprogramming cocktails consisting of multiple hepatic transcription factors [31]. We also demonstrated that the hepatic transdifferentiation procedure is a step-wise conversion process in which multiple molecular and cellular events occur in a sequential manner [31]. However, the mechanism underlying the generation of iHeps, including the role of each hepatic factor in the generation as well as maintenance of iHeps, is still largely unknown. To elucidate the role of *Hnf1a* in the maintenance of the reprogrammed hepatic state, we introduced the master hepatic factor *Hnf1a* [36–38] in mouse embryonic fibroblasts (MEFs) using an episomal vector system [13] in the presence of three small molecules, A83-01, BMP4, and CHIR99021 (ABR), as we had described previously [13, 31] (Figure 1(a)). On day 15 after transfection, we observed the first colonies with typical epithelial morphology expressing hepatic markers such as albumin, E-cadherin, and ZO-1 (Figure S1A). From the first epithelial colonies that we picked, we were able to generate stably expandable iHeps (Figures S1A and 2(b)). The *Hnf1a*-derived iHeps established using the episomal vector (hereafter referred to as 1a e-iHeps) expressed multiple hepatic markers at both the mRNA and protein levels, with complete inactivation of fibroblast markers (Figures 1(b)–1(d)). Notably, we were unable to detect the expression and integration of exogenous *Hnf1a* in the established 1a e-iHeps (Figures S1B and S1C), suggesting that 1a e-iHeps are indeed integration free. Nevertheless, a series of *in vitro* functional analyses demonstrated 1a e-iHeps had garnered key functional features to levels similar to iHeps reprogrammed by retroviral *Hnf1a* (1a r-iHeps) (Figures 1(e)–1(g)). Furthermore, 1a e-iHeps expressed multiple CYP450 enzymes as in 1a r-iHeps (Figure 1(h)), indicating that *Hnf1a* introduced by an episomal vector system is sufficient for inducing the direct conversion of MEFs into cells of a hepatic state.

### 3.2. Small Molecules Are Required for Maintaining 1a e-iHeps

**Figure 2:**
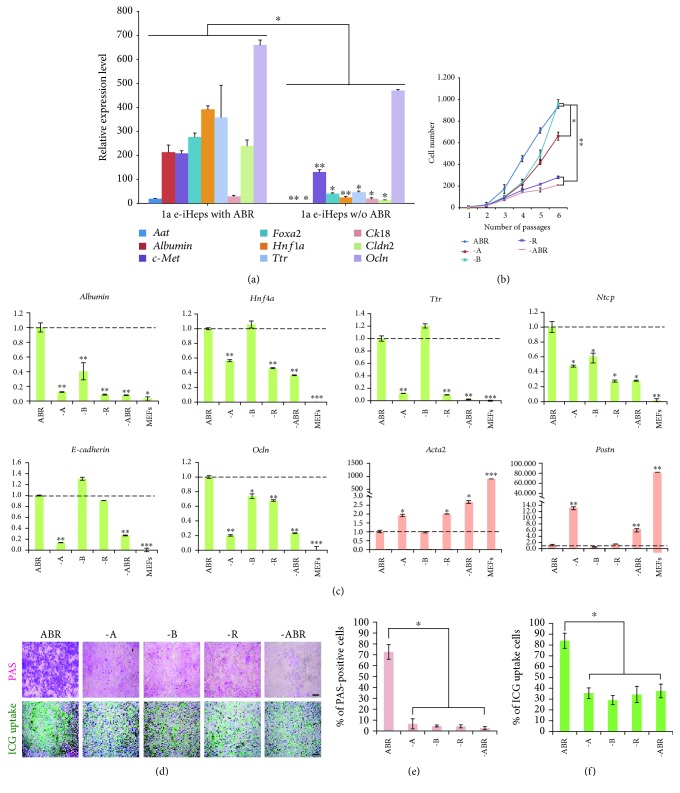
Small molecules support the maintenance of e-iHeps. (a) Relative gene expression levels of hepatocyte markers in e-iHeps in the presence or absence of small molecules. Error bars indicate the standard deviation of triplicate values. ^∗^ *P* < 0.05 and ^∗∗^ *P* < 0.01. (b) Proliferation rate of e-iHeps was measured after withdrawal of individual small molecules. The number of cells was calculated during serial passaging. Error bars indicate the standard deviation of triplicate values. ^∗^ *P* < 0.05 and ^∗∗^ *P* < 0.01. (c) Expression patterns of hepatocyte-specific (green) and fibroblast-specific (orange) markers were analyzed by qPCR after withdrawal of small molecules. Error bars indicate the standard deviation of triplicate values. ^∗^ *P* < 0.05, ^∗∗^ *P* < 0.01, and ^∗∗∗^ *P* < 0.001. (d) *In vitro* functional analysis of e-iHeps upon withdrawal of small molecules by PAS staining and ICG uptake. Scale bars: 100 *μ*m. (e and f) Percentage of PAS-positive (e) or ICG-positive (f) cells was measured. Error bars indicate the standard deviation of triplicate values. ^∗^ *P* < 0.05.

In our previous study [31], established 1a r-iHeps could be maintained stably even in the absence of small molecules upon successful conversion into a hepatic state, suggesting that small molecules play a limited role in the induction phase but not in the maintenance phase of the hepatic conversion process. To evaluate the effect of small molecule treatment (ABR) in the maintenance of the hepatic state driven by episomal *Hnf1a*, we examined the expression pattern of hepatic markers in the presence or absence of ABR. Upon withdrawal of ABR, 1a e-iHeps displayed strong downregulation of most hepatic markers (Figures 2(a) and S2A) with decreased cell proliferation (Figure 2(b)) as well as loss of typical hepatic cell morphology (Figure S2B), in contrast to 1a r-iHeps [31]. Furthermore, a number of fibroblast markers were dramatically upregulated upon withdrawal of ABR (Figure S2C). These results indicated that the reprogrammed state of 1a e-iHeps is not solid, unlike the case for 1a r-iHeps, and requires the continuous assistance of small molecules for maintaining the hepatic identity.

Next, we sought to determine which small molecule among ABR plays the most important role for maintaining the hepatic state of 1a e-iHeps. To this end, we omitted one small molecule at a time and looked at the effect. Upon withdrawal of A (A83-01) or R (CHIR99021), most hepatic markers were found to be rapidly and strongly downregulated (Figure 2(c)). In contrast, the omission of B (BMP4) produced a relatively milder decrease in hepatic gene expression (Figure 2(c)). However, in the absence of any one of the three small molecules, the *in vitro* ability for both glycogen storage and xenobiotic metabolism was severely impaired (Figures 2(d)–2(f)), indicating that all three small molecules are required for maintaining the hepatic state mediated by episomal *Hnf1a*.

### 3.3. Hnf1a-Mediated Hepatic State Is Metastable

**Figure 3:**
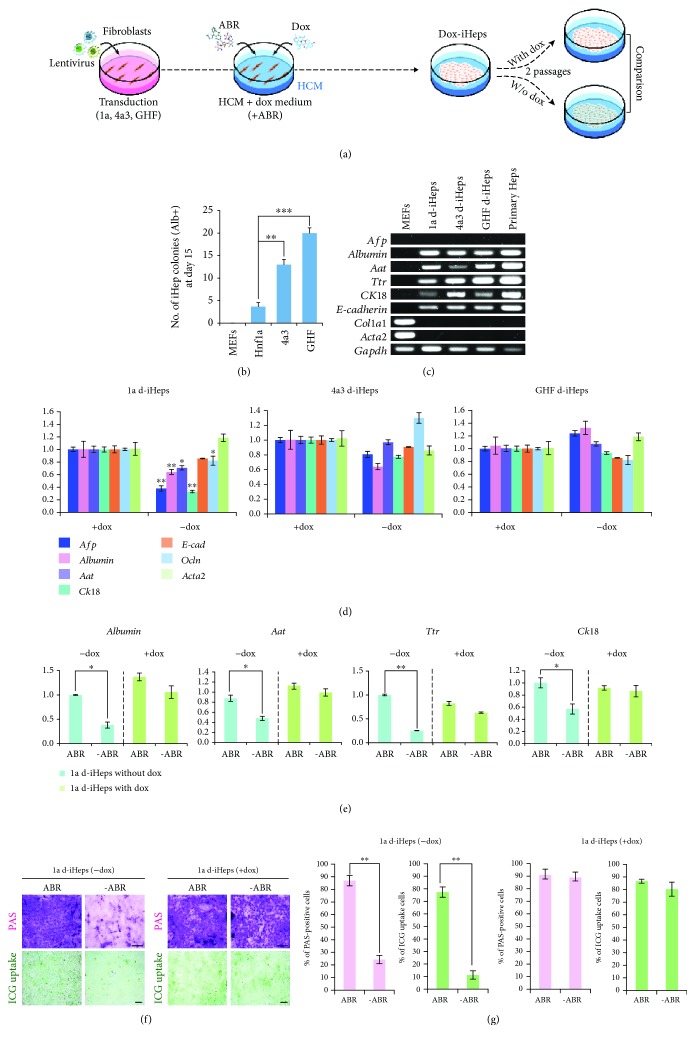
Generation of dox-inducible iHeps. (a) Schematic diagram representing the procedure of d-iHep generation by the dox-inducible gene delivery. (b) The number of iHep colonies expressing albumin was counted after 15 days of transduction. Error bars indicate the standard deviation of triplicate values. ^∗∗^ *P* < 0.01 and ^∗∗∗^ *P* < 0.001. (c) RT-PCR analysis describing the expression pattern of hepatocyte- and fibroblast-specific markers in the d-iHep lines. (d) Expression patterns of hepatocyte-specific markers in the d-iHep lines in the presence or absence of dox were compared by qPCR. Expression levels were normalized to d-iHeps cultured with dox. Error bars indicate the standard deviation of triplicate values. ^∗^ *P* < 0.05 and ^∗∗^ *P* < 0.01. (e) The expression pattern of hepatocyte markers in 1a d-iHeps in the presence or absence of dox and small molecules. Error bars indicate the standard deviation of triplicate values. ^∗^ *P* < 0.05 and ^∗∗^ *P* < 0.01. (f) *In vitro* functional analysis of 1a d-iHeps by PAS staining and ICG uptake in the presence or absence of both dox and small molecules. Scale bars: 100 *μ*m. Error bars indicate the standard deviation of triplicate values. ^∗^ *P* < 0.05. (g) Percentage of PAS- or ICG-positive cells was measured. Error bars indicate the standard deviation of triplicate values. ^∗^ *P* < 0.05.

As 1a r-iHeps did not lose their hepatic features even in the absence of small molecules [31], we next sought to investigate the role of exogenous *Hnf1a* in the maintenance of 1a r-iHeps. For this, we generated iHeps using a lentiviral vector encoding for *Hnf1a* under the control of a doxycycline- (dox-) inducible promoter (Figure 3(a)). Compared with MEFs transduced with multiple hepatic transprogramming factors, such as 4a3 (*Hnf4a* and *Foxa3*) and GHF (*Gata4*, *Hnf1a*, and *Foxa3*), *Hnf1a*-transduced MEFs exhibited relatively lower conversion efficiency as assessed by the number of iHep colonies (Figure 3(b)). The gene expression pattern and morphology of 1a d-iHeps (doxycycline iHeps) is comparable to those of 4a3 and GHF d-iHeps as well as primary hepatocytes (Figures 3(c), S3A, and S3B). Upon withdrawal of dox (Figure S3C), 1a d-iHeps exhibited downregulation of hepatic marker genes while both 4a3 and GHF d-iHeps were largely unaffected (Figure 3(d)). We checked the expression level of endogenous genes which are corresponding to reprogramming factors of 1a, 4a3, and GHF d-iHeps. Interestingly, upon withdrawal of dox, 1a d-iHeps showed drastic downregulation of endogenous *Hnf1a*, in contrast to 4a3 and GHF d-iHeps (Figure S3D), indicating that the continuous expression of an exogenous reprogramming factor is critical for maintaining the *Hnf1a*-mediated hepatic state. In contrast, exogenous reprogramming factors are dispensable after successful conversion of somatic cells into a hepatic state driven by multiple hepatic factors (Figure 3(d)).

**Figure 4:**
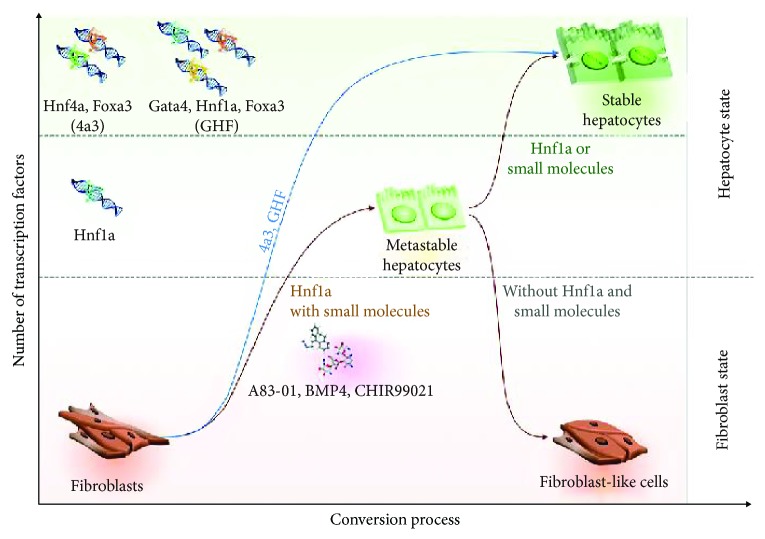
Graphical abstract describing the metastable hepatic state driven by *Hnf1a*. In contrast to the stable hepatic state driven by multiple hepatic factors (4a3 and GHF), the *Hnf1a*-mediated hepatic state is metastable and requires the continuous supply of either exogenous *Hnf1a* or small molecules.

Next, we investigated whether continuous treatment of 1a d-iHeps with small molecules, ABR, could stabilize the metastable state of 1a d-iHeps. To this end, we cultured 1a d-iHeps in the presence or absence of ABR (Figure 3(e)). 1a d-iHeps with dox exhibited stable expression of hepatic markers even in the absence of ABR; however, 1a d-iHeps without dox showed severely decreased expression upon withdrawal of ABR (Figure 3(e)). Notably, our functional assays also demonstrated that the *in vitro* ability for both glycogen storage and xenobiotic metabolism was severely impaired by withdrawal of both dox and small molecules (Figures 3(f) and 3(g)). Taken together, our data indicates that the *Hnf1a*-mediated hepatic state is metastable, requiring either continuous expression of exogenous *Hnf1a* or small molecules for maintaining hepatic cellular identity (Figure 4).

### 3.4. Discussion and Conclusion

Cell therapy is a potential treatment option for various hepatic diseases due to the shortage of donor livers [39, 40]. Although iPSC technology has long been considered as a patient-specific cell source for transplantable hepatocytes [41], safety issues concerning mainly the tumorigenic risk of iPSC-derived hepatocytes still preclude the clinical translation of these cells [42]. Recent studies have successfully demonstrated that specific sets of hepatic transcription factors could directly convert somatic cells into hepatocyte-like cells, namely, iHeps, without first generating an iPSC-like state [11–13, 31]. Furthermore, more flexible hepatic states such as bipotent hepatic stem cell-like cells or unipotent cholangiocyte progenitor-like cells could also be generated by direct conversion technology [10, 19]. However, the mechanism underlying the use of this technology for the direct conversion of somatic cells into those of a hepatic state is largely unknown.

In this study, we attempted to understand the mechanism of hepatic transprogramming by evaluating the role of exogenous reprogramming factors in both the initiation and maintenance phases of hepatic reprogramming. For this, we used our previous hepatic conversion strategy [31] in which the hepatic factor *Hnf1a* could sufficiently generate iHeps. Like in our previous study [31], introducing *Hnf1a* alone together with three small molecules (ABR) into MEFs using an episomal vector system was sufficient for generating iHeps (Figure 1). Moreover, the biological characteristics of 1a e-iHeps in terms of morphology (Figures S1A and S3A), marker expression (Figures 1(b) and S4), and functionality (Figures 1(f) and 1(g)) are highly similar with those of other 1a iHep lines, which are generated by different gene delivery systems (1a r-iHeps and d-iHeps). However, iHeps generated by episomal *Hnf1a* could not be maintained stably upon withdrawal of the small molecules (Figure 2), indicating that the hepatic state driven by episomal *Hnf1a* is not solid, unlike the case of iHeps reprogrammed by multiple hepatic transcription factors. To better elucidate the role of exogenous *Hnf1a* in the generation and maintenance of the iHep state, we next generated iHeps using dox-inducible *Hnf1a*. In contrast to iHeps generated by multiple hepatic factors, such as 4a3 and GHF, dox-inducible *Hnf1a*-mediated iHeps could not be maintained in the absence of both dox and small molecules (Figure 3). Collectively, our findings indicate that the *Hnf1a*-mediated hepatic state is metastable, requiring either continuous expression of exogenous *Hnf1a* or small molecules for stabilizing the metastable hepatic state of iHeps (Figure 4).

To investigate the underlying mechanism of metastable reprogramming status of *Hnf1a*-derived iHeps, we compared the activation of endogenous hepatic genes using different reprogramming factor combinations. For this, we measured the expression patterns of endogenous hepatic genes in iHep lines generated by distinct reprogramming cocktails (1a, 4a3 and GHF d-iHeps) in the absence or presence of dox (Figure S3D). Interestingly, the 1a d-iHeps could not maintain the endogenous expression of *Hnf1a* without either continuous expression of exogenous *Hnf1a* (Figure S3D) or support of small molecules (Figure 2(a)). These results indicate that the singular expression of *Hnf1a* is not enough to stably reset epigenetic status of iHeps for maintaining hepatocyte-specific transcriptome. The stable hepatic state of iHeps might be achieved by transient expression of additional reprogramming factor such as *Foxa3,* a well-known pioneer factor which can modulate epigenetic status of target genomic regions [43, 44].

Direct conversion technology has been considered an alternative to iPSC technology [1, 2, 45, 46] due to its relatively simple and fast procedure [31] that results in the direct conversion of somatic cells into cell types with nontumorigenic properties [10, 25, 31, 47, 48] when using cell type-specific transcription factors [10, 25, 31, 42, 47, 48]. To translate this technology to the clinic, many previous studies have tried to screen for the minimum number of transcription factors in combination for inducing specific cellular identities [9–31]. Indeed, recent studies have demonstrated the direct conversion of somatic cells into specific functional cell types using even a single transcription factor, i.e., *Oct4* for iPSCs [49, 50], either *Sox2* or *Oct4* for induced neural stem cells (iNSCs) [14, 18], *Oct4* for induced oligodendrocytes (iOPCs) [16], *Oct4* for induced cardiomyocytes (iCMs) [17], *Ascl1* for induced neurons (iNs) [15], and finally *Hnf1a* for iHeps [31]. However, it has not been clearly addressed whether the reprogramming state driven by a single reprogramming factor is equally stable to those mediated by multiple reprogramming factors.

We had previously showed that *Hnf1a*-mediated iHeps are superior to iHeps generated by multiple hepatic factors in terms of gene expression pattern and *in vitro* functionality [31]. However, in the current study, we show that a single hepatic factor-mediated hepatic state is metastable and that hepatic states driven by multiple hepatic factors are more stable, indicating that a systematic interaction between each reprogramming factor is important for conferring a stable and functional cellular identity onto fibroblasts. Thus, for further translating this direct conversion strategy to the clinic, we should carefully screen for not just the minimum number of transcription factors but also the combination of factors needed for successfully converting somatic cells into functional cell types with a stable reprogramming state. Moreover, directly converted cell types should be carefully assessed for transgene dependence, as both the uncontrolled expression of exogenous transgenes and the continuous transgene dependence of reprogrammed cell types may inhibit the potential application of this technology such as *in vivo* direct conversion.

## Data Availability

The data used to support the findings of this study are included within the article and supplementary information file.

## Supplementary Materials

Supplementary Materials Figure S1: generation of e-iHeps using the hepatic transcription factor Hnf1a. Figure S2: loss of hepatic features in e-iHeps upon withdrawal of small molecules. Figure S3: generation of d-iHeps using different transcription factor cocktails. Figure S4: comparative analysis among 1a iHep lines derived from distinct gene delivery systems. Table S1: primers used for RT-PCR and qPCR.
