# Supplementary material for: Metastable Reprogramming State of Single Transcription Factor-Derived Induced Hepatocyte-Like Cells
Source: Stem Cells Int. 2019 Apr 7;2019:6937257. doi: 10.1155/2019/6937257 (PMC6476006; doi:10.1155/2019/6937257)
Supplement: Supplementary Materials — Figure S1: generation of e-iHeps using the hepatic transcription factor Hnf1a. Figure S2: loss of hepatic features in e-iHeps upon withdrawal of small molecules. Figure S3: generation of d-iHeps using different transcription factor cocktails. Figure S4: comparative analysis among 1a iHep lines derived from distinct gene delivery systems. Table S1: primers used for RT-PCR and qPCR. [file 6937257.f1.docx]

**­Supplementary Content**

**Metastable reprogramming state of single transcription factor–derived induced hepatocyte-like cells**

Seon In Hwang, Tae Hwan Kwak, Ji Hyun Kang, Jonghun Kim, Hyunseong Lee, Kee Pyo Kim, Kinarm Ko, Hans R. Schöler, and Dong Wook Han

**Supplementary figures**

**Figure S1, Figure S2, Figure S3, Figure S4**

**Supplementary table**

**Table S1**

**Supplemental information**

**Supplementary Figure S1**

**
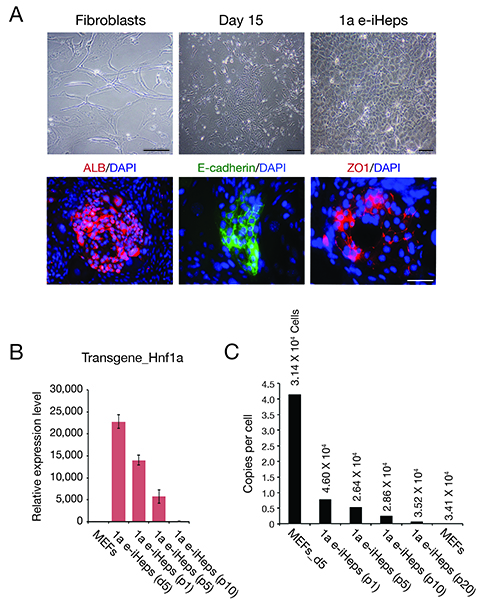
**

**Figure S1. Generation of e-iHeps using the hepatic transcription factor Hnf1a.** (**A**) Morphological changes during e-iHep generation as assessed by bright-field images (upper panel). Immunocytochemistry analysis of the first e-iHep colonies expressing Albumin, E-cadherin, and ZO-1. Scale bars, 100 μm. (**B**, **C**) Gradual decrease of exogenous *Hnf1a* expression (**B**) and copy number of the intracellular episomal cassettes (**C**) in e-iHeps during serial passaging. Numbers (**C**) indicate the cell numbers estimated in each sample. MEFs on day 5 after transfection were used as a positive control.

**Supplementary Figure S2**

**
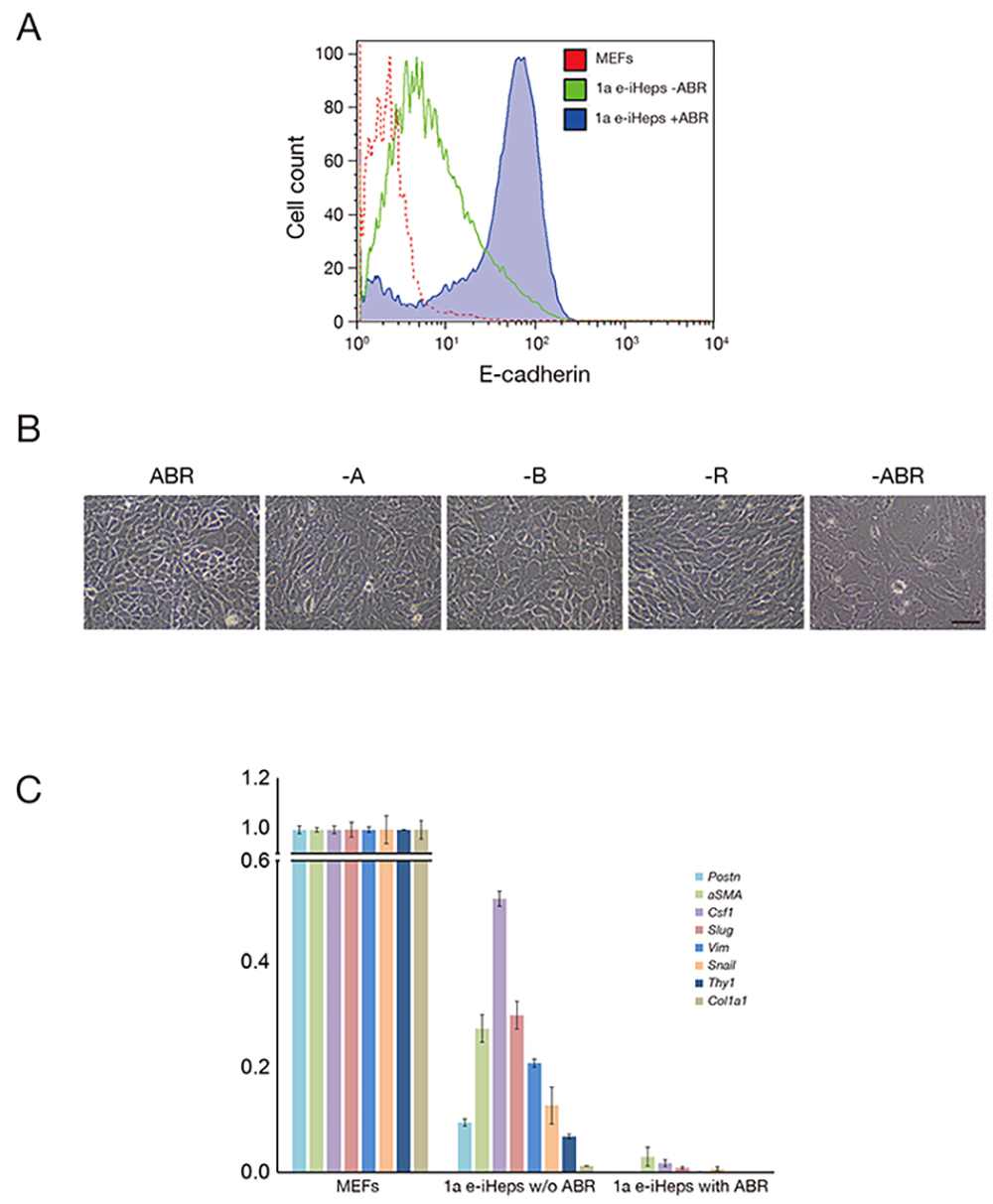
**

**Figure S2. Loss of hepatic features in e-iHeps upon withdrawal of small molecules.** (**A**) The proportion of E-cadherin-positive cells after withdrawal of small molecules was measured by flow cytometry analysis. (**B**) The morphology of e-iHeps after withdrawal of each small molecule. Scale bars, 100 μm. (**C**) The relative expression level of fibroblast-specific genes in 1a e-iHeps upon withdrawal of ABR. Expression levels were normalized to those of MEFs.

**Supplementary Figure S3**


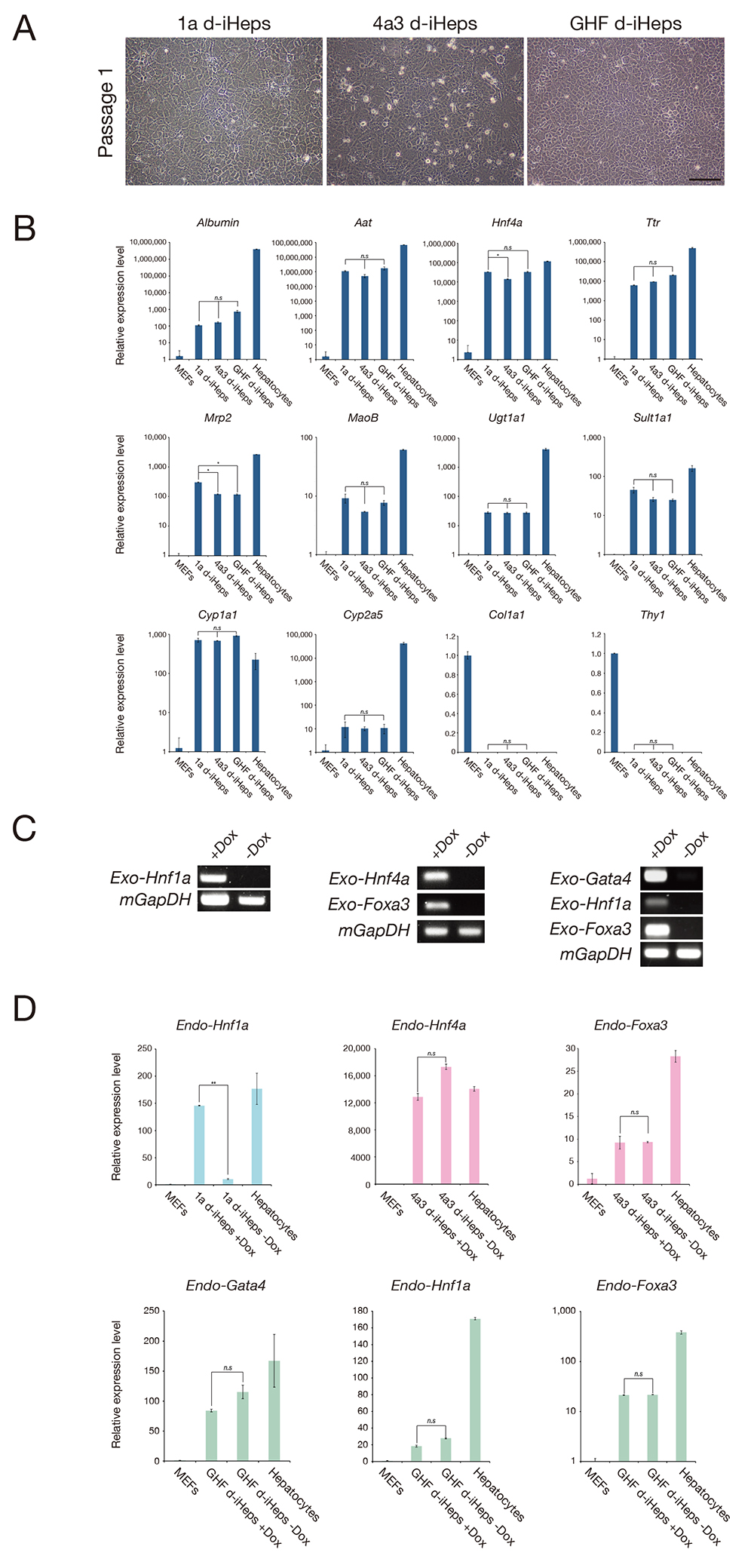


**Figure S3. Generation of d-iHeps using different transcription factor cocktails.** (**A**) Morphology of established d-iHep lines generated by distinct transcription factor combinations (Hnf1a, 4a3, and GHF). Scale bars, 100 μm. (**B**) Expression patterns of hepatocyte- and fibroblast-specific markers in d-iHep lines derived from distinct reprogramming factors. Error bars indicate the standard deviation of triplicate values. **P*<0.05. (**C**) Expression of transgenes in the d-iHep lines (Hnf1a, 4a3, and GHF) in the presence or absence of dox was analyzed by RT-PCR. (**D**) Relative gene expression level of endogenous genes which are corresponding to reprogramming factors of 1a, 4a3 and GHF d-iHeps in the presence or absence of dox. Error bars indicate the standard deviation of triplicate values. **P*<0.05, ***P*<0.01.

**Supplementary Figure S4**

**
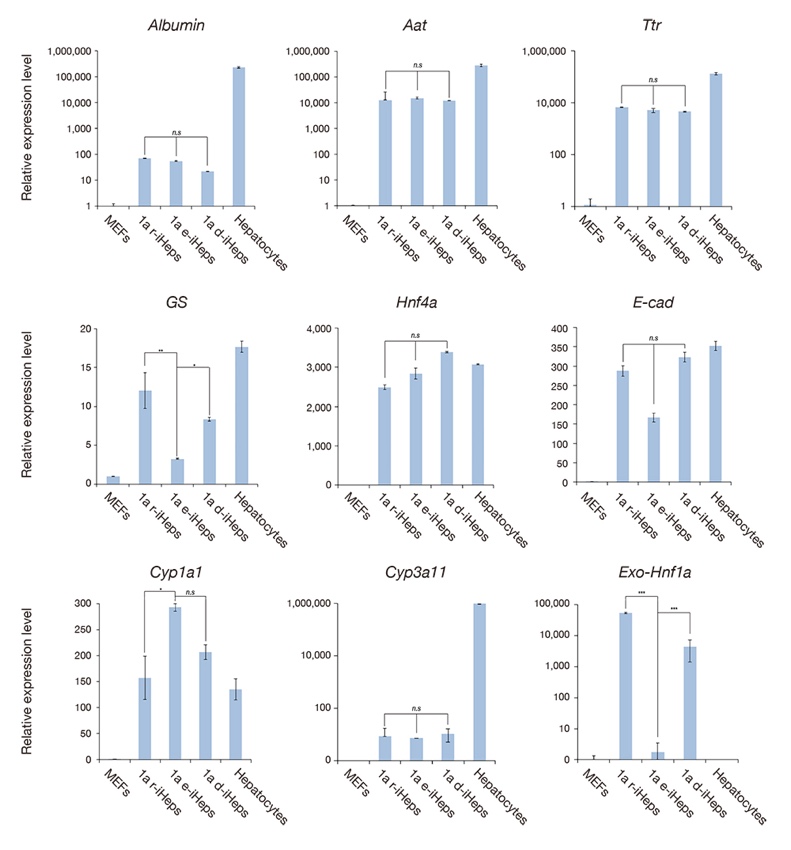
**

**Figure S4. Comparative analysis among 1a iHep lines derived from distinct gene delivery systems.** Expression patterns of hepatocyte-specific markers in iHep lines derived from distinct reprogramming systems (retroviral vector, episomal vector and dox-inducible lentiviral vector). Error bars indicate the standard deviation of triplicate values. **P*<0.05, ***P*<0.01, ****P*<0.001.

**Supplementary Table S1. Primers used for RT-PCR and qPCR.**

| **Gene Name** | **Genebank Number** | **Primer sequence** | |
| --- | --- | --- | --- |
| *Afp* | NM_007423 | 5’- CGTGATGCTTTGGGCGTTTA -3’ |  |
|  |  | 5’- GCCAAAAGGCTCACACCAAAG -3’ |  |
| *Alb* | NM_009654 | 5’- AAACCTTGTCACTAGATGCAAAGACG -3’ |  |
|  |  | 5’- GGGTAGCCTGAGAAGGTTGTGG -3’ |  |
| *Hnf1a* | NM_009327 | 5’- CCTGCTGCCATCCAACCATA -3’ |  |
|  |  | 5’- CCACGGTTACTGGGAAGAGGA -3’ |  |
| *Hnf4a* | NM_008261 | 5’- GCCAACGATCACCAAGCAAG -3’ |  |
|  |  | 5’- TGAGGGTATGAGCCAGCAGAA -3’ |  |
| *Ttr* | NM_013697 | 5’- CCCTGCTCAGCCCATACTCCTA -3’ |  |
|  |  | 5’- TGCTTTGGCAAGATCCTGGT -3’ |  |
| *CK18* | NM_010664 | 5’- GATCGTGGATGGCAGAGTGG -3’ |  |
|  |  | 5’- TTCCCTCCTTCTCTGCCTCAGT -3’ |  |
| *NTCP* | NM_001177561 | 5’- TTCAAGAAGCTGGCGGACAT -3’ |  |
|  |  | 5’- CATCTCCCATGGTGCCACAC -3’ |  |
| *Foxa2* | NM_010446 | 5’- CCTGGCTGCAGACACTTCCTAC -3’ |  |
|  |  | 5’- CAGGGCCTGAAAGCCATCTT -3’ |  |
| *E-cadherin* | NM_009864 | 5’- TTCAAGAAGCTGGCGGACAT -3’ |  |
|  |  | 5’- CATCTCCCATGGTGCCACAC -3’ |  |
| *Ocln* | NM_008756 | 5’- TCGCACATCAAGAGGATGGTG -3’ |  |
|  |  | 5’- GCCTCTGGAGAGAATTGCAGAGA -3’ |  |
| *Cldn2* | NM_016675 | 5’- TCTGCTCAACAGCCCAAAGC -3’ |  |
|  |  | 5’- TGGTTCTTCACACATACCCAGTCA -3’ |  |
| *Acta2* | NM_007392 | 5’- ATCGTCCACCGCAAATGCTT -3’ |  |
|  |  | 5’- AACTGGAGGCGCTGATCCAC -3’ |  |
| *Col1a1* | NM_007742 | 5’- CCCTGCCTGCTTCGTGTAAA -3’ |  |
|  |  | 5’- TCGTCTGTTTCCAGGGTTGG -3’ |  |
| *Postn* | NM_015784 | 5’- TCAAGGGCCTAGAAGACGATCA -3’ |  |
|  |  | 5’- AAACTCTGTGGTCTGGCCTCTG -3’ |  |
| *Thy1* | NM_009382 | 5’- CTTTCCCTCTCCCTCCTCCAAG -3’ |  |
|  |  | 5’- CGAGGGCTCCTGTTTCTCCTT -3’ |  |
| *Csf1* | NM_007778 | 5’-CTGACCCAGGATGAGGACAGAC -3’ |  |
|  |  | 5’-AGTCCTGTGTGCCCAGCATAGA -3’ |  |
| *Slug* | NM_011546 | 5’- TCCAGAATGTCGCTTCTGCAT-3’ |  |
|  |  | 5’-GCATTCTGTTTGAGTAAACACTGGTTG-3’ |  |
| *Snail* | NM_011427 | 5’-GCACTTGAACTTGCGGTTTCC -3’ |  |
|  |  | 5’-CCTTGCTCCACAAGCACCAA -3’ |  |
| *Vim* | NM_13574311 | 5’-CCTCTGGTTGACACCCACTCA -3’ |  |
|  |  | 5’-TCCTTCTTGCTGGTACTGCACTG -3’ |  |
| *Gapdh* | NM_008084 | 5’- CCAATGTGTCCGTCGTGGAT -3’ |  |
|  |  | 5’- TGCCTGCTTCACCACCTTCT -3’ |  |
| *Cyp1a1* | NM_009992 | 5’- CCTTCCGGCATTCATCCTTC -3’ |  |
|  |  | 5’- TTTCAGGCCGGAACTCGTTT -3’ |  |
| *Cyp2a5* | NM_007812 | 5’- GCACTTCCTAGATGACAAGGGACA -3’ |  |
|  |  | 5’- CAGGCTCAACGGGACAAGAA -3’ |  |
| *Cyp2d22* | NM_001163472 | 5’- CCTCTCCTCGGCTGAGTTTCA -3’ |  |
|  |  | 5’- CGCCAGTGCATCAGGTTCA -3’ |  |
| *Cyp3a11* | NM_007818 | 5’- TTCCAGCCTTGTAAGGAAACACA -3’ |  |
|  |  | 5’- TGTACTGAATCTTTAACCAGGCATCA -3’ |  |
| *Cyp3a13* | NM_007819 | 5’- TCCTGCAGAACTTCACTGTCCA -3’ |  |
|  |  | 5’- TGGTTTCTGGTCCACAGGATACA -3’ |  |
| *Fbxo15* | NM_015798 | 5’- ATGGCCACGTGGAGAGAGG -3’ |  |
|  |  | 5’- TGCTGTGACACTGAACTCCCTTC -3’ |  |
| *GSc* | NM_010351 | 5’- CGCCTGGGCTACAACAGCTA -3’ |  |
|  |  | 5’- CCGGAGACACCAGTACAGAACC -3’ |  |
| *Mrp2* | NM_013806 | 5’- ACTTTCAATGCCGGCTTCCT-3’ |  |
|  |  | 5’- TGGTCCTAGACAGCGGCAAG-3’ |  |
| *MaoB* | NM_172778 | 5’- GTGGGCCAGGAAACCAAGAG-3’ |  |
|  |  | 5’- TTTGGCAGCCAGAACCAGAA-3’ |  |
| *Ugt1a1* | NM_201645 | 5’- CTGGAGCTCGCGTTTGCTT-3’ |  |
|  |  | 5’- CCTTCTGCTGGAGCTGCTGA-3’ |  |
| *Sult1a1* | NM_133670 | 5’- CCATGGCTAACTACACAACCATCC-3’ |  |
|  |  | 5’- CACAGCTAAGGAGAGGACCCTTG-3’ |  |
| *episomal-Hnf1a* | | 5’- GATGGCCTCCTCTTCCCAGA -3’ |  |
|  |  | 5’- AGGCATTAAAGCAGCGTATCCA -3’ |  |
| *OriP/EBNA-1* | | 5’- CATCATCATCCGGGTCTCCA -3’ |  |
|  |  | 5’- CAGTGCTTGGGCCTTCTCCT -3’ |  |
